# CARD9 Is Required for Classical Macrophage Activation and the Induction of Protective Immunity against Pulmonary Cryptococcosis

**DOI:** 10.1128/mBio.03005-19

**Published:** 2020-01-07

**Authors:** Althea Campuzano, Natalia Castro-Lopez, Amanda J. Martinez, Michal A. Olszewski, Anutosh Ganguly, Chrissy Leopold Wager, Chiung-Yu Hung, Floyd L. Wormley

**Affiliations:** a Department of Biology, South Texas Center for Emerging Infectious Diseases, The University of Texas at San Antonio, San Antonio, Texas, USA; b Division of Pulmonary and Critical Care Medicine, Department of Internal Medicine, Michigan Medicine University, Ann Arbor, Michigan, USA; c VA Ann Arbor Healthcare System, Research Service, Ann Arbor, Michigan, USA; d Division of Hepatobiliary Surgery, Department of Surgery, Michigan Medicine, University of Michigan, Ann Arbor, Michigan, USA; University of Michigan Medical School

**Keywords:** CARD9, *Cryptococcus neoformans*, cryptococcosis, fungal immunology, fungal pathogenesis, host-pathogen interactions, innate immunity, macrophages, medical mycology, pattern recognition receptors

## Abstract

Caspase recruitment domain-containing protein 9 (CARD9) is a critical adaptor molecule triggered by the interaction of C-type lectin receptors (CLRs) with carbohydrate motifs found in fungi. Consequently, clinical and animal studies indicate that CARD9 is an important regulator of protective immunity against fungal pathogens. Previous studies suggest that CARD9 is important for the induction of protection against Cryptococcus neoformans, an opportunistic fungal pathogen that causes life-threatening infections of the central nervous system in immunocompromised patients. However, the effect of CARD9 deficiency on the induction of protective immune responses against C. neoformans is unknown. Immunization with a C. neoformans mutant that overexpresses the transcription factor zinc finger 2, denoted LW10, results in protection against an otherwise lethal challenge with wild-type (WT) C. neoformans. Our results showed that CARD9 is essential for the induction of vaccine-mediated immunity against C. neoformans infection. We observed significant decreases in interleukin-17 (IL-17) production and significant increases in Th2-type cytokine (IL-4, IL-5, and IL-13) production in CARD9-deficient mice after inoculation with strain LW10. While leukocyte infiltration to the lungs of CARD9-deficient mice was similar in LW10 and WT C. neoformans-infected mice, macrophages derived from CARD9-deficient mice inherently skewed toward an M2 activation phenotype, were unable to contain the growth of LW10, and failed to produce nitric oxide in response to infection with LW10 or stimulation with lipopolysaccharide. These results suggest that CARD9-mediated signaling is required for M1 macrophage activation and fungicidal activity necessary for the induction of vaccine-mediated immunity against C. neoformans.

## Abstract (precis)

Cryptococcus neoformans is a fungal pathogen that is found throughout the environment and can cause life-threatening infections of the lung and central nervous system in severely immunocompromised individuals. Caspase recruitment domain-containing protein 9 (CARD9) is a critical molecule that is activated after interactions of C-type lectin receptors (CLRs) found on the surfaces of specific immune cells, with carbohydrate structures associated with fungi. Patients with defects in CARD9 are significantly more susceptible to a multitude of fungal infections. C. neoformans contains several carbohydrate structures that interact with CLRs on immune cells and activate CARD9. Consequently, these studies evaluated the necessity of CARD9 for the induction of protective immunity against C. neoformans infection. These results are important, as they advance our understanding of cryptococcal pathogenesis and host factors necessary for the induction of protective immunity against C. neoformans.

## INTRODUCTION

Cryptococcus neoformans, the predominant etiological agent of cryptococcosis, is an opportunistic fungal pathogen globally distributed throughout the environment. The primary route of infection is via inhalation of desiccated basidiospores or budding yeast. *Cryptococcus* can be cleared from the lungs, remain dormant in lung alveoli, or can disseminate to the central nervous system (CNS). Dissemination to the CNS, bones, or other tissues occurs in patients with severe deficiencies in CD4^+^ T cell-mediated immunity, resulting in 15% of AIDS-related global deaths each year (1, 2). However, *Cryptococcus* is also capable of affecting individuals with no obvious underlying immunological deficiencies as demonstrated in the damage response framework by Pirofski and Casadevall (3). Therefore, effective clearance during the first encounter with the yeast is critical in preventing dissemination of the pathogen. We rely on resident pulmonary phagocytic cells, namely, macrophages and dendritic cells (DCs), to identify and effectively eradicate *Cryptococcus.* Phagocytic cells identify incoming pathogens through their highly conserved pattern recognition receptors (PRRs), such as Toll-like receptors (TLRs) and C-type lectin receptors (CLRs) and NOD-like receptors (NLRs); however, the key PRR(s) required for the recognition of *Cryptococcus* remains to be elucidated (4).

Caspase recruitment domain containing protein 9 (CARD9) is a critical adaptor molecule that operates downstream of CLRs and is present within myeloid cells. CARD9 forms a complex with B cell lymphoma 10 (BCL10) and mucosa-associated lymphoid tissue lymphoma translocation protein 1 (MALT1) for the induction of canonical nuclear factor kappa beta (NF-κB) and mitogen-activated protein kinase (MAPK) activation. Activation of NF-κB and MAPK triggers macrophage activation, DC maturation, and cytokine production necessary for the induction of protection against fungal pathogens (5). Yamamoto and colleagues determined that defects in CARD9 results in impaired control of Cryptococcus deneoformans strain B3501 infection in mice possibly due to defective Th1- and Th17-type cytokine production (6). Recently, Huang and colleagues demonstrated increased susceptibility to various cryptococcal species by CARD9-deficient mice compared to wild-type (WT) mice (7). However, the role of CARD9 during the induction of protective immune responses against *Cryptococcus* has not been elucidated.

LW10 or P*_GPD1_*-*ZNF2* is a C. neoformans mutant that overexpresses the transcription factor zinc finger 2 (Znf2), resulting in filamentation and avirulence in mice (8). Zhai and colleagues demonstrated that immunization of mice with strain LW10 results in the induction of Th1 and Th17 cell-mediated immune responses and protection against an otherwise lethal pulmonary infection by wild-type C. neoformans (9). Consequently, we endeavored to utilize LW10 within an experimental murine model of cryptococcosis to determine the role of CARD9 in mediating protection against pulmonary C. neoformans infections.

Our results show that CARD9 is essential for the induction of protective immune responses against C. neoformans. Although CARD9 does not impact recruitment of phagocytes during the induction phase of vaccination, CARD9-deficient mice exhibited significant decreases in interleukin-17 (IL-17) cytokine production and significant increases in Th2-type cytokine and chemokine production in the lungs after immunization with strain LW10. Pulmonary macrophages of CARD9-deficient mice preferentially polarized toward a nonprotective M2 macrophage activation phenotype. Additionally, CARD9-deficient macrophages demonstrated an inherent defect in the production of nitric oxide, resulting in an inability of CARD9-deficient mice to control replication and dissemination of the attenuated LW10 strain. These results show that CARD9 is required for M1 macrophage polarization and the induction of protective immune responses against pulmonary C. neoformans infection.

## RESULTS

### CARD9 is required for protective antifungal immunity.

**FIG 1:**
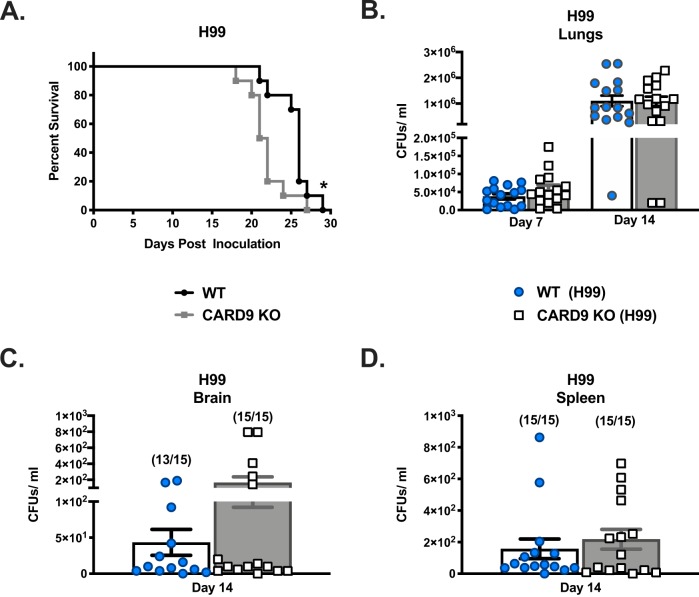
WT and CARD9 mice are both highly susceptible to the C. neoformans H99 strain. (A to D) C57BL/6 and CARD9-deficient mice were given an intranasal inoculation with C. neoformans strain H99. (A) Mice were observed for 29 days for survival analysis. Survival data are from one experiment using 10 mice per group, and the data was analyzed using the log rank test. (B) Pulmonary fungal burden was determined on day 7 or 14 postinoculation. (C and D) Fungal burden was determined in the brains (C) and spleens (D) of mice on day 14 postinoculation. Fungal burden data show the cumulative results of three experiments using five mice per group per time point. Bars indicate the means ± standard errors of the means (SEM) (error bars). Values that are significantly different (*P* < 0.05) by an unpaired Student’s *t* test (two-tailed test) or log rank test compared to the values for CARD9-deficient mice are indicated by an asterisk.

**FIG 2:**
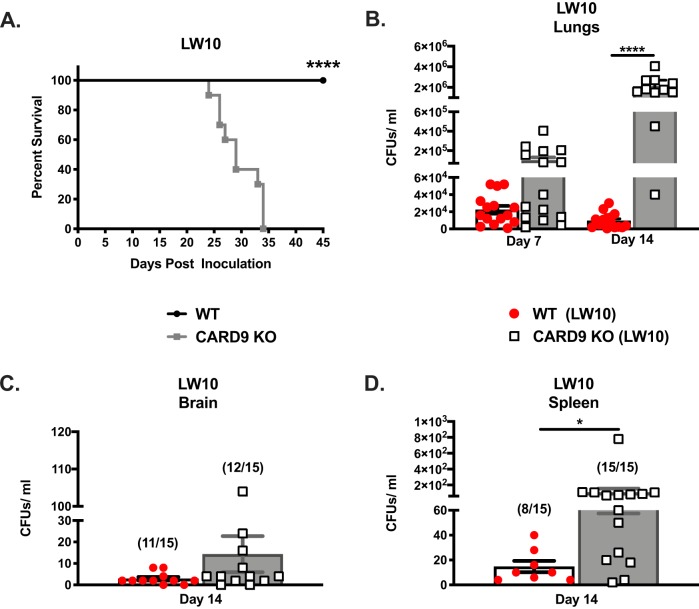
CARD9 is required for the induction of protection against cryptococcosis. (A to D) C57BL/6 and CARD9-deficient mice were given an intranasal inoculation with C. neoformans*-*derived strain LW10. (A) Mice were observed for up to 45 days for survival analysis. (B to D) Next, pulmonary fungal burden was determined on day 7 or 14 postinoculation (B), or fungal burden was determined in the brains (C) and spleens (D) of mice on day 14 postinoculation. Survival data are from one experiment using 10 mice per group. Fungal burden data are cumulative results for three experiments using five mice per group per time point. Values that are significantly different from the values for CARD9-deficient mice by the (A) log rank test or (B to D) an unpaired Student’s *t* test (two-tailed test) are indicated by asterisks as follows: *, *P* < 0.05; ****, *P* < 0.0001.

The impact of CARD9 on the induction of protective immune responses against fungi has been characterized in endemic mycosis; however, the role of CARD9 during the induction of vaccine-mediated immunity to *Cryptococcus* has yet to be determined (10). Consequently, we investigated the overall impact of CARD9 deficiency on the induction of protective antifungal immune responses using a murine model of pulmonary cryptococcosis. To do this, CARD9-deficient and wild-type (WT) mice were given a pulmonary infection with WT C. neoformans strain H99 or a C. neoformans strain overexpressing *ZNF2* (LW10). Strain LW10 is avirulent in immunocompetent mice, and immunization with LW10 protects mice from a subsequent otherwise lethal pulmonary infection with C. neoformans strain H99 cells (9). Survival (morbidity) was monitored for 30 or 45 days postinoculation (Fig. 1A and Fig. 2A), while pulmonary fungal burden was evaluated in a separate group of infected mice on days 7 and 14 postinoculation (Fig. 1B to D and Fig. 2B to D). CARD9-deficient mice demonstrated significantly higher susceptibility to pulmonary C. neoformans H99 infection compared to WT mice (*P* = 0.03, median survival of 21 and 26 days postinfection for WT and CARD9 knockout [KO] mice, respectively; Fig. 1A). No significant differences were observed in the pulmonary fungal burden of CARD9-deficient mice compared to WT mice with C. neoformans strain H99 at days 7 and 14 postinoculation (Fig. 1B). Previous studies indicated that CARD9 deficiency results in increased dissemination of fungal pathogens to the CNS (11). However, we noted no significant difference in fungal burdens in both the brains and spleens of WT mice, compared to CARD9-deficient mice, at day 14 postinfection (Fig. 1C and D).

WT and CARD9-deficient mice received a higher inoculum of strain LW10, 10^6^ cells/mouse, to be in accordance with previous studies demonstrating the efficacy of LW10 to induce protective immunity against pulmonary cryptococcosis (9). Figure 2A shows that CARD9-deficient mice were significantly more susceptible to pulmonary LW10 infection than WT mice (*P* < 0.0001) with a median survival time of 29 days. All WT mice infected with C. neoformans strain LW10 were alive and appeared healthy upon termination of the experiment at day 45 postinoculation. We observed significant increases in the pulmonary fungal burden of strain LW10 in CARD9-deficient mice, compared to WT mice, at day 14 postinfection (Fig. 2B; *P* < 0.05) and noted the presence of this trend toward increasing LW10 burden in CARD9-deficient mice at day 7 postinfection. Furthermore, we observed a significant increase in the fungal burden in the splenic tissues of CARD9-deficient mice, compared to WT mice, at day 14 postinfection (Fig. 2D; *P* < 0.05). No significant difference in brain fungal burden was observed in CARD9-deficient mice compared to WT mice. These results indicate that CARD9 is required for the induction of protective immune responses against pulmonary C. neoformans infections.

### CARD9 deficiency results in fungal growth unrestrained by pulmonary leukocytes.

We evaluated whether CARD9 deficiency alters the leukocyte profile observed following inoculation with strain LW10. To do this, we used flow cytometry to quantify pulmonary leukocyte recruitment in CARD9-deficient and WT mice following immunization with strain LW10. Additionally, we determined the profile of leukocyte infiltrates observed in C. neoformans strain H99 infected CARD9-deficient and WT mice as gated in Fig. S1 in the supplemental material. Pulmonary leukocytes were isolated from enzymatically dispersed lungs of WT and CARD9-deficient mice on days 7 and 14 postinoculation with C. neoformans strain H99 or LW10. We observed no statistically significant differences in total CD45^+^ leukocyte infiltration between WT and CARD9-deficient mice infected with either strain on day 7 and 14 postinoculation (Fig. S2). Furthermore, no significant differences were observed in all pulmonary leukocyte subsets tested, including interstitial macrophages, alveolar macrophages, dendritic cells (DCs), B cells, polymorphonuclear neutrophils (PMNs), eosinophils, CD4^+^ T cells, CD8^+^ T cells, NK cells, NK T cells, plasmacytoid DC (pDCs), and γδ T cells between WT and CARD9-deficient mice (Fig. S2).

10.1128/mBio.03005-19.1 FIG S1 Gating strategy for pulmonary leukocytes. Download FIG S1, PDF file, 0.7 MB. Copyright © 2020 Campuzano et al. 2020 Campuzano et al. This content is distributed under the terms of the Creative Commons Attribution 4.0 International license.

10.1128/mBio.03005-19.2 FIG S2 CARD9 deficiency does not impact pulmonary leukocyte infiltration during cryptococcosis. Total leukocyte infiltration was evaluated at days 7 and 14 postinoculation in C57BL/6 and CARD9 KO mice following infection with C. neoformans strain H99 or LW10. Lungs were excised and analyzed for pulmonary infiltrates. Leukocytes were labeled with anti-CD45 antibodies or dually labeled with anti-CD45 and antibodies for specific cells and analyzed by flow cytometry. Data shown are the means ± SEM of absolute cell numbers from three independent experiments performed using five mice per group per time point. Download FIG S2, PDF file, 0.1 MB. Copyright © 2020 Campuzano et al. 2020 Campuzano et al. This content is distributed under the terms of the Creative Commons Attribution 4.0 International license.

**FIG 3:**
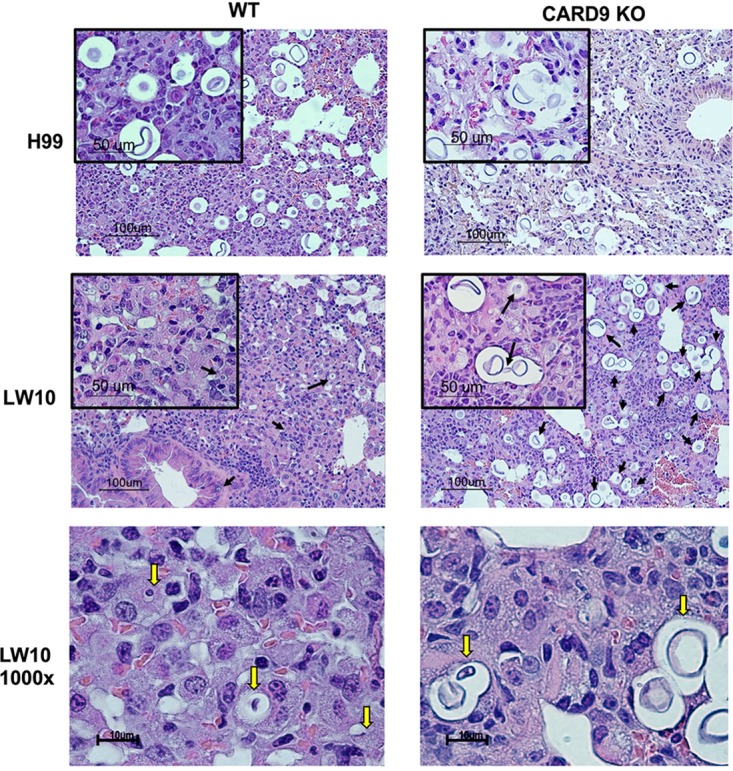
CARD9 deficiency leads to unchecked fungal growth. C57BL/6 and CARD9-deficient mice were given an intranasal inoculation with 1 × 10^4^ CFU of C. neoformans strain H99 (top row) or 1 × 10^6^ CFU of LW10 (middle row). The lungs of H99- or LW10-infected C57BL/6 mice and CARD9-deficient mice were collected on day 7 postinoculation and processed for histopathological analysis. Images were taken using 10× objectives (100 μm). The small panel is 40× (50 μm) of the original 10× objective power. The 100× objective was exclusively used to observe the degradation process of *Cryptococcus* by leukocytes exposed to LW10. During an acute pulmonary infection, C57BL/6 and CARD9-deficient mice are unable to control fungal burden, which allows the proliferation and expansion of *Cryptococcus* and the formation of titan cells. Alternatively, C57BL/6 mice inoculated with the attenuated LW10 strain effectively controlled fungal growth and prevented the proliferation of yeast at day 7 postinoculation. In contrast, CARD9-deficient mice allow for progression of fungal growth and subsequent pathogenesis. At high-power images, we observe *Cryptococcus* (yellow arrows) undergoing degradation following internalization by phagocytic cells in C57BL/6 mice (bottom left). CARD9-deficient mice allow for proliferation of *Cryptococcus* (yellow arrows) and formation of large titan cells (bottom right). Images are representative images derived from two independent experiments using four mice per group (two male and two female mice per group).

Although CARD9 deficiency did not alter the leukocyte recruitment profile following infection with strain LW10, we performed a histological examination of lung tissue sections prepared from WT and CARD9-deficient mice inoculated with either C. neoformans strain H99 or LW10. The lungs of both WT and CARD9-deficient mice infected with C. neoformans strain H99 displayed increased fungal burden and budding of C. neoformans yeast (Fig. 3, top row). The WT mice inoculated with strain LW10 were able to control cryptococcal growth and prevent titan cell formation (Fig. 3, middle row, left panel). In contrast, CARD9-deficient mice were unable to control fungal growth and were unable to contain yeast cells with the titan cell morphology in the H99-infected group (Fig. 3, top row, right panel) as well as in the LW10-infected group of mice (Fig. 3, middle row, right panel, and bottom row). Analysis of histological sections from LW10-infected mice under high-power magnification revealed significant differences in cellular processing of yeast in WT mice compared to CARD9-deficient mice (Fig. 3, bottom row). Ingested LW10 within phagocytic cells of WT mice appear to be in various stages of degradation, matching the significantly diminished fungal burden shown in Fig. 2. In contrast, abundant, enlarged, and seemingly proliferating LW10 yeast cells were prevalent in the lung sections of CARD9-deficient mice. Furthermore, the enlarged size and the morphology of the yeast suggested the development of titan cells in CARD9-deficient mice.

**FIG 4:**
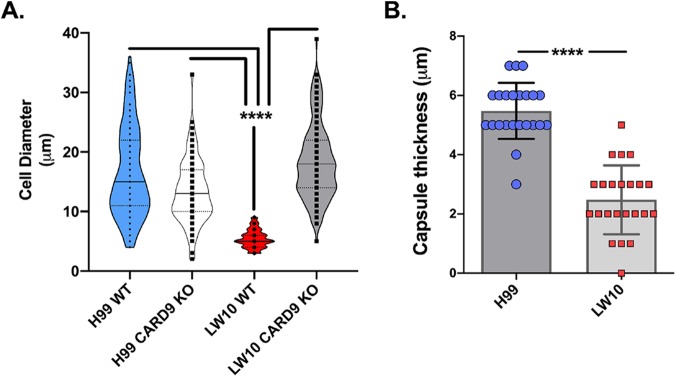
CARD9 deficiency in LW10-infected mice promote LW10 titan cell generation but does not fully restore the defect in capsule formation. (A and B) Outcomes of morphometric analysis for cryptococcal cell diameter (A) and capsule size (B) in H&E-stained lung sections. Note that the titan cells are not being formed by the LW10 strain in the WT mice but are robustly produced in the CARD9 KO mice (A). The LW10 strain is capable of capsule induction in CARD9 KO mice, but the capsule size is substantially reduced (B). There were four mice in each group. Values that are significantly different (*P* < 0.0001) by two-way ANOVA with Dunnett correction are indicated by bars and four asterisks.

Titan cells are resistant to engulfment by phagocytic cells due to their enlarged size and exhibit immunomodulatory properties (12–14). Thus, we performed a morphometry study (Fig. S3A) to evaluate the frequencies of *Cryptococcus* titan cells (>10 μm in size) in all four groups of infected mice. Results show no major differences in the diameter of C. neoformans strain H99 in CARD9-deficient mice compared to WT mice, with 75% or more yeast cells displaying the enlarged dimensions of titan cells (Fig. 4A). In contrast, LW10 yeast cells in WT mice were ingested by macrophages demonstrating features of degradation (Fig. 3, bottom row). Even the apparently intact LW10 population had significantly smaller diameter, on average 5 μm, and virtually no cells displayed the dimensions or morphology of titan cells (Fig. 4A). However, CARD9 deficiency enabled titan cell formation by the LW10 strain, where titan cells became the dominant population in the infected lungs, similarly to the H99 strain infections (Fig. 4A and Fig. S3B). We also performed measurements of the capsule size formed around the H99 and LW10 yeasts in the infected lungs (Fig. S3A). The capsule surrounding the fungal cell wall of strain LW10 in WT mice could not be reliably measured due to its partial degradation and the uneven thickness around the perimeter of the cells. Thus, the morphometric comparison was restricted to CARD9-deficient mice (Fig. 4B). The capsule size produced by LW10 cells was significantly reduced (on average by half; *P* < 0.0001) despite the fact that the LW10 cell size and frequency of titan cells were not diminished relative to those of H99 cells. Thus, the defect in LW10 capsule production persists even in the presence of defective host response, as observed in the CARD9-deficient mice and is independent of LW10’s ability to produce titan cells. Together, these data support that CARD9 is necessary for immune activation of phagocytic cells and is required for their antifungal activity against C. neoformans.

10.1128/mBio.03005-19.3 FIG S3 Morphometry measures of fungal cell and capsule sizes. (A) Measurements for cell diameter (blue marks) and capsule size (red marks) were conducted in images of the lung sections and calculated and performed at the randomized fields (*n* = 4 mice per group). (B) Frequency plot illustrates distribution of diameters sorted using frequency distribution analysis tool in Prism 8.1 in 2-μm increment bins and illustrated as histograms. Green dotted area represents cell size range consistent with titan cells. Download FIG S3, PDF file, 0.4 MB. Copyright © 2020 Campuzano et al. 2020 Campuzano et al. This content is distributed under the terms of the Creative Commons Attribution 4.0 International license.

### CARD9 deficiency alters the pulmonary cytokine profile during C. neoformans infection.

Despite observing no significant differences in leukocyte infiltration, the histological evaluation suggested that CARD9 deficiency results in increased fungal burden and an inability of phagocytes to effectively kill *Cryptococcus*. Experimental pulmonary infection of A/J mice with strain LW10 results in enhanced IL-17 and gamma interferon (IFN-γ) production by CD4^+^ T cells that decreases as the infection is resolved (9). Therefore, we sought to investigate the impact of CARD9 deficiency on pulmonary cytokine and chemokine production following inoculation with strain LW10 (Table 1).

**TABLE 1:** CARD9 deficiency leads to nonprotective cytokine responses during pulmonary cryptococcosis

Cytokine	Cytokine level (pg/ml)^*a*^							
	H99				LW10			
	Day 7		Day 14		Day 7		Day 14	
	WT	CARD9 KO	WT	CARD9 KO	WT	CARD9 KO	WT	CARD9 KO
Th1-type								
IL-2	60.57 ± 5.04	66.97 ± 16.26	149.41 ± 9.01	60.04 ± 14.94	36.04 ± 4.72	33.98 ± 3.16	20.76 ± 10.92	37.05 ± 8.89
IFN-γ	57.25 ± 1.67	53.06 ± 11.71	66.35 ± 25.74	130.66 ± 25.10	48.26 ± 3.37	50.78 ± 1.88	48.36 ± 23.84	62.06 ± 14.64
IL-12p40	117.03 ± 23.47	61.78 ± 21.38	***340.81 ± 79.22***	***349.28 ± 63.79***	203.88 ± 48.59	192.84 ± 31.37	***575.13 ± 201.50***	***596.76* ± *176.88***
IL-12p70	113.94 ± 45.06	98.72 ± 16.43	261.65 ± 93.86	***346.93 ± 97.25***	107.84 ± 24.88	108.86 ± 39.24	**376.98 *+* 78.09**	101.51 ± 70.80

Proinflammatory								
IL-1α	51.39 ± 15.34	49.12 ± 16.15	***791.51 ± 25.20***	***135.29 ± 37.09***	110.21 ± 30.50	50.66 ± 17.95	121.83 ± 47.86	***135.99 ± 38.14***
IL-1β	21.40 ± 2.08	19.0 ± 3.94	***48.38 ± 12.09***	***35.90 ± 13.35***	38.82 ± 4.88	33.96 ± 9.60	23.64 ± 10.08	28.40 ± 14.11
TNF-α	194.78 ± 53.45	123.59 ± 37.19	121.61 ± 27.10	225.52 ± 23.00	260.35 ± 33.9	166.24 ± 8.22	***92.09 ± 30.13***	64.55 ± 23.00
IL-6	25.51 ± 6.79	29.66 ± 3.55	43.84 ± 3.48	58.66 ± 5.22	22.80 ± 2.81	40.90 ± 6.74	17.07 ± 7.05	**345.80 ± 15.92**
IL-17	12.79 ± 1.68	7.44 ± 1.87	***27.91 ± 2.52***	12.97 ± 2.80	**61.64 ± 5.77**	38.82 ± 3.43	**17.42 ± 9.03**	9.20 ± 2.87

Th2-type								
IL-4	53.77 ± 1.33	36.84 ± 0.70	***164.37 ± 41.18***	***156.51 ± 34.84***	45.26 ± 6.31	119 ± 2.02	61.33 ± 1.75	**290.29 ± 15.79**
IL-5	13.22 ± 1.88	12.98 ± 0.98	***38.17 ± 8.29***	***36.99 ± 8.82***	9.58 ± 2.39	18.42 ± 1.78	19.72 ± 6.02	***168.61*** ± ***10.43***
IL-13	218.53 ± 43.37	148.60 ± 34.02	***395.98 ± 155.28***	***349.98 ± 184.86***	181.32 ± 68.85	204.54 ± 12.59	280.27 ± 130.02	**2,619.79 ± 173.11**
IL-10	26.81 ± 3.42	18.17 ± 4.43	33.80 ± 20.17	36.42 ± 18.88	21.07 ± 4.43	31.57 ± 8.42	24.70 ± 18.88	28.59 ± 16.95

Chemokines								
G-CSF	45.32 ± 8.72	22.56 ± 8.72	50.38 ± 7.77	32.29 ± 8.42	29.07 ± 4.91	31.07 ± 4.49	10.52 ± 7.90	**26.27 ± 8.42**
GM-CSF	12.97 ± 8.05	14.96 ± 5.78	17.27 ± 9.75	12.29 ± 5.78	12.29 ± 5.78	12.29 ± 6.55	12.10 ± 8.27	16.02 ± 9.51
MCP-1 (CCL2)	353.58 ± 25.06	478.30 ± 28.04	***697.94*** ± ***67.23***	***781.6*5 ± *64.82***	412.79 ± 44.12	**923.27 ± 26.55**	298.89 ± 72.02	**2,100.81 ± 69.63**
MIP-1α (CCL3)	133.87 ± 4.18	259.86 ± 3.60	**1,308.49 ± 14.64**	***944.92 ± 9.89***	439.22 ± 57.66	844.70 ± 4.22	611.27 ± 50.52	**2,192.72 *+* 44.11**
MIP-1β (CCL4)	55.96 ± 9.93	45.07 ± 6.69	**394.52 ± 44.93**	***106.45 ± 27.73***	73.48 ± 5.98	100.10 ± 31.39	71.59 ± 49.01	272.90 ± 30.99
RANTES (CCL5)	348.40 ± 81.43	259.31 ± 67.91	***1,040.38 ± 349.12***	***1,738.89 ± 284.84***	919.24 ± 332.69	1,213.24 ± 74.27	1,794.60 ± 564.98	1,245.47 ± 326.07
Eotaxin (CCL11)	363.59 ± 110.87	420.89 ± 131.99	***1,205.58 ± 264.64***	***1,566.26 ± 284.28***	968.71 ± 304.16	**2,128.09 ± 234.68**	746.52 ± 231.76	**2,362.96 ± 661.90**
KC (CXCL1)	94.81 ± 10.7	90.59 ± 6.48	**202.13 *+* 12.17**	65.50 ± 10.11	205.79 ± 35.93	113.16 ± 10.90	115.48 ± 26.79	50.12 ± 14.99

a Cytokines were analyzed in C57BL/6 and CARD9 KO mice inoculated with C. neoformans strain H99 or LW10 at day 7 or 14 postinfection as indicated in the table. Data shown are expressed as means ± SEM and are the cumulative results for two experiments utilizing five mice per group. Boldface values indicate a significant increase (*P* < 0.05) comparing the WT value to the CARD9 KO value at the same time point. Italic boldface values indicate a significant increase comparing each group (WT or CARD9 KO) at day 7 to a similar group at day 14 (*P* < 0.05) by the Kruskal-Wallis test with Dunn’s multiple-comparison test.

Lung homogenates were prepared from WT and CARD9-deficient mice on days 7 and 14 postinoculation with C. neoformans strain H99 or LW10 and analyzed for Th1-associated (IL-2, IFN-γ, IL-12p40, and IL-12p70), Th2-associated (IL-4, IL-5, IL-13, and IL-10), Th17-associated (IL-17), and proinflammatory (IL-1α, IL-1β, tumor necrosis factor alpha [TNF-α], and IL-6) cytokines and chemokines (granulocyte colony-stimulating factor [G-CSF], granulocyte-macrophage colony-stimulating factor [GM-CSF], monocyte chemotactic peptide 1 [MCP-1]/CC chemokine ligand 2 [CCL2], macrophage inflammatory protein 1α [MIP-1α]/CCL3, MIP-1β, CCL4, RANTES [*r*egulated on *a*ctivation, *n*ormal *T* cell *e*xpressed and *s*ecreted]/CCL5, Eotaxin/CCL11, keratinocyte-derived chemokine [KC]/CXC chemokine ligand 1 [CXCL1]) levels (Table 1). Among these cytokines, we observed significant increases in IL-17, CCL3, CCL4, and CXCL1 production on day 14 postinoculation in WT mice infected with C. neoformans strain H99 compared to CARD9-deficient mice infected with H99 (boldface values). We noted an overall increase in proinflammatory, Th1-type and Th2-type cytokine and chemokine levels in mice infected with C. neoformans strain H99 when comparing day 7 to day 14 values. We also observed significantly increased production of IL-17 on days 7 and 14 postinoculation, and we observed increased IL-12p70 levels on day 14 postinoculation in WT mice infected with LW10 compared to pulmonary cytokine levels observed in CARD9-deficient mice inoculated with LW10. On the other hand, murine CARD9 deficiency significantly increased levels of IL-6, multiple Th2-associated cytokines (IL-4, IL-5, and IL-13) and several chemokines (G-CSF, CCL2, CCL3, and CCL11) in pulmonary homogenates prepared from CARD9-deficient mice on day 14 postinoculation compared to WT mice inoculated with LW10, consistent with a “dysregulated” immune response. Studies indicated that a predominant Th2-type cytokine milieu is associated with nonprotective anticryptococcal immune responses, disease progression, and increased susceptibility to pulmonary cryptococcosis (15–18). Together our data identified that CARD9 deficiency results in diminished Th17 and increased Th2-type cytokine production and nonprotective immune responses against pulmonary C. neoformans infection. Additionally, the chemokine production appears to not be compromised in CARD9-deficient mice and enhanced, presumably as a compensatory response to the increased pulmonary fungal burden.

### Macrophage anticryptococcal activity is impaired in the absence of CARD9.

**FIG 5:**
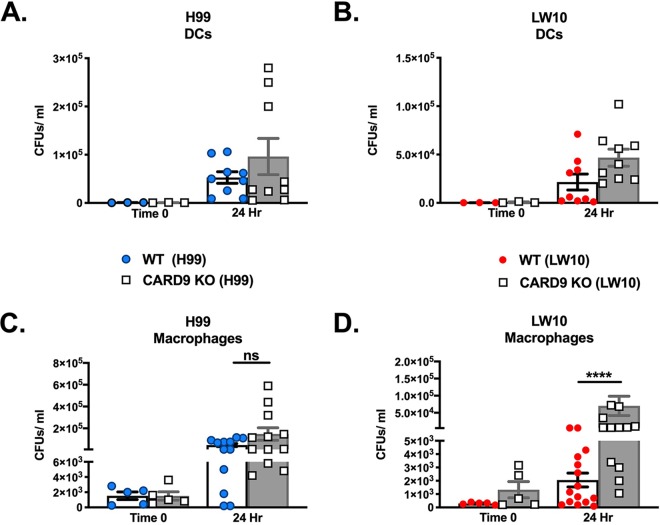
CARD9-deficient macrophages are unable to effectively kill cryptococci. (A to D) Pulmonary DCs (A and B) or macrophages (C and D) were isolated from C57BL/6 and CARD9-deficient mice on day 7 postinoculation and immediately processed to quantify fungal burden or cultured *ex vivo* for 24 h and then lysed with endotoxin-free deionized water to enumerate the internalized cryptococci. Data shown are the means ± SEM and are cumulative of five independent experiments using three or five mice per group. Statistical significance by an unpaired Student’s *t* test (two-tailed test) is indicated as follows: ****, *P* < 0.0001; ns, not significant.

Our evaluation of pulmonary leukocyte infiltrates in infected mice did not reveal any significant quantitative differences in leukocyte subpopulations to account for the inability of LW10 to elicit protective immune responses in CARD9-deficient mice, while pathological studies suggest a defect in LW10 killing by phagocytes. Therefore, we sought to evaluate whether any qualitative differences in the antifungal activity of specific phagocyte populations, specifically DCs and pulmonary macrophages, may explain the lack of protection observed in CARD9-deficient mice. We elected not to evaluate antifungal activity of neutrophils due to previous studies indicating that neutrophils are either not required or are detrimental to the host during pulmonary cryptococcosis (19, 20). To investigate the direct anticryptococcal activity of pulmonary phagocytes in mice lacking CARD9, DCs and macrophages were isolated from WT and CARD9-deficient mice infected with either C. neoformans strain H99 or LW10 at 7 days postinfection. The cells were diluted to the appropriate cell density and then either immediately lysed to determine the fungal burden within each cell type at the time of sacrifice or incubated *ex vivo* and then lysed to release any intracellular cryptococci. This allowed us to ascertain the capacity of each phagocyte population to contain cryptococcal growth (Fig. 3A to B). We observed no significant differences in the anticryptococcal activity of DCs from WT or CARD9-deficient mice against strain H99 (Fig. 5A) or LW10 (Fig. 5B). Also, no significant differences were observed in the anticryptococcal activity of macrophages from WT or CARD9-deficient mice, as they were each unable to contain the growth of H99 following 24 h of incubation *ex vivo* to a similar extent (Fig. 3C). In contrast, macrophages isolated from CARD9-deficient mice infected with LW10 were unable to control cryptococcal growth following 24 h of incubation *ex vivo* compared to macrophages isolated from WT mice (Fig. 5D, *P* < 0.0001). These results demonstrate that pulmonary macrophages require CARD9 for optimal antifungal activity.

### Macrophages from CARD9-deficient mice are skewed toward an M2 phenotype.

**FIG 6:**
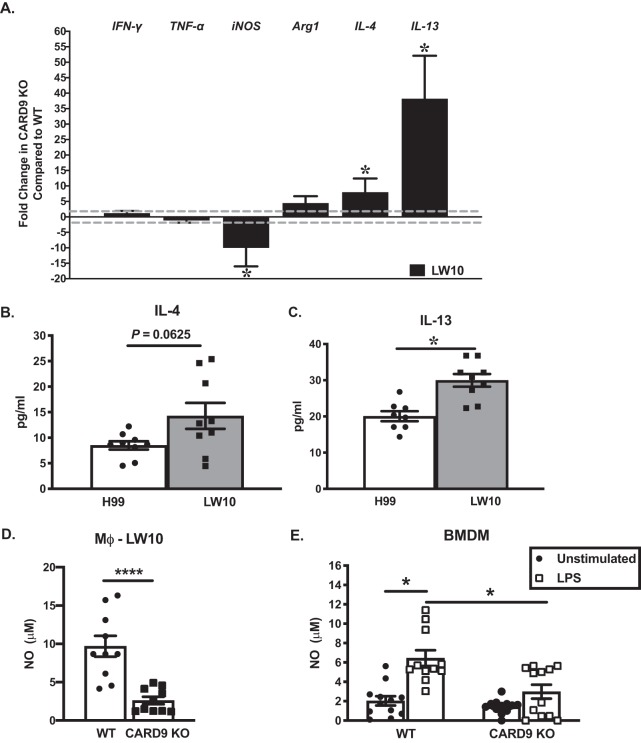
CARD9 deficiency in pulmonary macrophages result in M2 phenotype. C57BL/6 mice were given an intranasal inoculation with C. neoformans strain LW10. Pulmonary F4/80^+^ macrophages were isolated from the lungs at day 7 postinoculation. (A) Total RNA was extracted, and transcripts were analyzed for M1-associated markers (IFN-γ, NOS2, and TNF-α) and M2-associated markers (arginase-1 [Arg1], IL-4, and IL-13). Dashed lines indicate the twofold change cutoff comparing results obtained from macrophages of CARD9 KO mice to results derived from C57BL/6 pulmonary macrophages. The indicated mRNA levels were normalized to GAPDH. Results are expressed as the means plus SEM and are cumulative of at least three experiments using five mice per group. (B and C) Macrophages (Mϕ) were cultured *ex vivo* for 24 h, and IL-4 (B) and IL-13 (C) cytokine levels and nitric oxide levels were determined from supernatants. Results are expressed as means ± SEM and are cumulative of at least three independent studies with three biological replicates. Significantly different values are indicated by asterisks as follows: *, *P* < 0.05; ****, *P* < 0.0001. An unpaired Student’s *t* test (two-tailed test) was used to test data in panels B to D, and Kruskal-Wallis test with Dunn’s multiple-comparison test was used to test data in panel E.

Macrophage polarization has a critical role in either aiding in fungal clearance or allowing the proliferation and subsequent dissemination of C. neoformans and is thus predictive of disease outcome. Classically activated macrophages (M1) are critical for the eradication of *Cryptococcus*, whereas alternatively activated macrophages (M2) allow cryptococcal proliferation and survival, resulting in exacerbation of disease (21–24). Therefore, to examine macrophage polarization, we quantified the expression of genes associated with M1 and M2 macrophage activation in macrophages isolated from the lungs of CARD9-deficient mice compared to WT mice on day 7 postinoculation. We observed no significant differences in mRNA transcripts or cytokines associated with either M1 or M2 macrophage polarization by pulmonary macrophages from mice inoculated with H99. C. neoformans strain H99 is known to induce M2 polarization of pulmonary macrophages; therefore, we focused our efforts on elucidating the role of CARD9 in the presence of LW10. Pulmonary F4/80^+^ macrophages were isolated, and total RNA was evaluated for the expression of genes commonly associated with macrophage activation using real-time PCR (Fig. 4A). Conversely, we observed a significant decrease in the expression of the M1 marker inducible nitric oxide synthase (iNOS) in macrophages from CARD9-deficient macrophages of mice inoculated with LW10 compared to macrophages from WT mice (*P* < 0.05; Fig. 6A). Furthermore, gene expression of M2 macrophage markers IL-4 and IL-13 were significantly upregulated in CARD9-deficient macrophages compared to WT macrophages (*P* < 0.05; Fig. 6A). Gene expression for cytokines IL-4 and IL-13 are associated with a nonprotective Th2-type response and M2 macrophage polarization (25). Altogether, the gene expression profiling results suggest that macrophages from CARD9-deficient mice inoculated with LW10 attain an M2 polarization phenotype. Next, we isolated F4/80^+^ macrophages from the lungs of CARD9-deficient and WT mice infected with LW10 on day 7 postinoculation. The macrophages were cultured for 24 h, and the culture supernatant was collected to assay for IL-4 and IL-13 cytokine levels (Fig. 6B and C). We did observe a trend toward more IL-4 production by macrophages from CARD9-deficient mice infected with LW10 (*P* = 0.0625) compared to macrophages from WT mice infected with LW10 (Fig. 4B). Furthermore, CARD9-deficient macrophages enriched from LW10-inoculated mice produced significantly increased IL-13 levels compared to macrophages from WT mice infected with LW10 (*P* < 0.05; Fig. 6A). M2 macrophage polarization is typically observed in the lungs of mice following experimental pulmonary infection with C. neoformans strain H99 (26). These results indicated that in the absence of CARD9, macrophages are primed to be even more skewed toward an M2 activation phenotype which is associated with exacerbation of fungal infections compared to their WT macrophage counterparts.

We also analyzed nitric oxide (NO) levels in the culture supernatant of macrophages isolated from CARD9-deficient and WT mice infected with LW10 on day 7 postinoculation. NO production by M1 polarized macrophages is a critical component of their antifungal activity, particularly in the context of eliciting protective anticryptococcal responses (27). We observed significantly less NO production in culture supernatant of macrophages from CARD9-deficient mice infected with LW10 compared to pulmonary macrophages derived from WT mice infected with LW10 on day 7 postinoculation (*P* < 0.0001; Fig. 6D). Subsequently, we cultured bone marrow-derived macrophages (BMDMs) from WT or CARD9-deficient mice in the presence or absence of lipopolysaccharide (LPS) to compare the potential of macrophages from CARD9-deficient mice to produce NO. BMDMs derived from WT mice produced significantly more NO compared to unstimulated BMDMs from WT mice (*P* < 0.05; Fig. 6E) and BMDMs from CARD9-deficient mice cultured in the presence or absence of LPS (*P* < 0.05 for each condition; Fig. 6E). Our results indicate that CARD9-deficient BMDMs stimulated with LPS produced NO at levels equivalent to those observed for unstimulated BMDMs from WT or CARD9-deficient mice, further suggesting that CARD9 is essential for M1 macrophage activation and the induction of protective immunity against C. neoformans.

## DISCUSSION

Our results demonstrated that signaling via the adaptor molecule CARD9 is required for the induction of protective immunity against pulmonary cryptococcosis. Studies by the research group of Xiaorong Lin determined that morphological transition is linked to virulence and effective cryptococcal clearance (8). C. neoformans strain LW10 constitutively overexpresses cryptococcal morphogenesis regulator, zinc finger 2 (*Znf*2), resulting in hyphal formation both *in vitro* and *in vivo* (8). *Cryptococcus* is not considered a classical dimorphic fungus; however, *Cryptococcus* is capable of filamentation that is required for mating (8, 28). Hyphenating C. neoformans is effectively eradicated by the host response, demonstrating that filamentation comes at a cost of virulence loss (29). Mice previously immunized with LW10 were protected from cryptococcosis following an otherwise lethal challenge with WT C. neoformans compared to mock-immunized mice (9). Since we noted that LW10 induces protective responses in mice, we were particularly interested to examine the role of CARD9 in the context of protective immunity against cryptococcosis. When we utilized CARD9-deficient mice, we noted that protection against LW10 strain could no longer be achieved. We show that CARD9-deficient mice inoculated with LW10 demonstrate significant increases in pulmonary fungal burden and dissemination to splenic tissues and succumbed to infection with LW10, whereas WT mice were able to resolve the infection. We did not note significant differences in dissemination to the brain which has been historically observed in the Znf2 overexpression strain XL280, resulting in attenuation of virulence and reduced fungal burden in the brain but not in other organs (30). This suggests that Znf2 interferes with cryptococcal neurotropism during extrapulmonary dissemination (30). Although we did not observe any significant differences in leukocyte infiltrates between CARD9-deficient and WT mice immunized with LW10, histological analysis of lung tissues from CARD9-deficient mice also revealed deficiencies in fungal clearance. We also noted significantly increased titan cell formation in the CARD9-deficient mice. Generation of cryptococcal titan cells has been demonstrated both *in vivo* and *in vitro* (31, 32). One of the most significant features of titan cells is their enlarged size (>10 μm) relative to typical cryptococcal cells ranging between 5 and 7 μm in diameter. Titan cells have been shown to enhance dissemination in the host, as they are unable to be phagocytized due to their large size and they also produce dense capsules (14). The density of the capsule can affect permeability to complement proteins (13). Furthermore, we observed a significant decrease in IL-17A production in the lungs of CARD9-deficient mice compared to WT mice. CARD9 mutations are associated with defects in Th17-type cell immune responses in CARD9-deficient patients (33–35), although experimental studies show that while beneficial, IL-17A is not absolutely essential for protection against pulmonary cryptococcosis (6, 19, 36). Th2-type cytokines IL-4, IL-5, and IL-13, which are associated with disease exacerbation, were significantly increased at day 14 postinoculation in CARD9-deficient mice immunized with LW10. The levels of chemokines CCL2, CCL3, and CCL11 were significantly increased in CARD9-deficient mice, indicating that CCL chemokine production does not require CARD9 signaling and may reflect a greater need for leukocyte trafficking in mice in response to the increased pulmonary fungal burden. Overall, our data point out that the immune response upon CARD9 deficiency is dysregulated and ineffective for fungal eradication.

Experimental studies with mice demonstrated that CARD9 plays an essential role in protection against fungal pathogens, including Candida albicans, Cryptococcus neoformans, and Aspergillus fumigatus (6, 11, 37). Yamamoto and colleagues first evaluated the role of CARD9 during an acute pulmonary infection with *C. deneoformans* (formally known as serotype D) and noted significant increases in fungal burden, as well as decreased Th1 and Th17-type responses in CARD9-deficient mice compared to WT mice (6). Studies utilizing an experimental model of pulmonary cryptococcosis in which mice received intratracheal inoculations with *Cryptococcus* serotype A, B, C, D or an AD hybrid, demonstrated that CARD9-deficient mice were susceptible to the fungal infections at an early time point (7). CARD9 autosomal recessive defects is associated with increased susceptibility to fungal infections, including, but not limited to, greater susceptibility to chronic mucocutaneous candidiasis, phaeohyphomycosis, and dermatophytic disease (38, 39). The roles of CLRs during cryptococcosis are of interest, as the majority of studies demonstrate that CLRs are needed for the recognition of carbohydrate moieties and host defense against several clinically relevant fungal pathogens (37, 40–46). The initial recognition and host response to the inhalation of *Cryptococcus* in the lungs are typically the responsibility of pulmonary macrophages and dendritic cells that are constantly surveying the lung alveoli for pathogen-associated molecular patterns (PAMPs) through their germ line encoded pattern recognition receptors (PRRs). PAMP recognition results in the activation of antimicrobial host defenses and stimulation of the adaptive immune response (18, 47). Thus, we determined the antimicrobial activity of DCs and pulmonary macrophages against *Cryptococcus* in this model system. We chose not to evaluate the antimicrobial activity of neutrophils due to previous studies in our lab and others showing either no or an even deleterious role of neutrophils during the protective immune responses to pulmonary cryptococcosis (19, 20). Although DCs did not require CARD9 to contain fungal burden, we noted that pulmonary macrophages required CARD9 signaling for their antifungal activity. We demonstrated that CARD9 deficiency had a significant deleterious impact toward skewing pulmonary macrophages toward a nonprotective M2 phenotype, expressing more arginase-1, IL-4, and IL-13 compared to WT pulmonary macrophages. *Cryptococcus* drives a nonprotective immune response that is associated with Th2-type cytokine production and alternative, or M2, macrophage activation resulting in the inability of the host to clear the yeast (15, 17, 48, 49). In contrast, protective immune responses against cryptococcosis are associated with Th1-type cytokine responses and classical, or M1, macrophage activation (M1) (17, 50, 51). Previous studies by our lab indicated the critical role for nitric oxide in the anti-*Cryptococcus* activity of macrophages (27). Macrophages derived from CARD9-deficient mice did not produce nitric oxide while infected with LW10 or following LPS stimulation compared to macrophages derived from WT mice. These studies indicate that macrophages require CARD9 signaling for optimal anti-*Cryptococcus* activity and nitric oxide production. Previous studies suggested that the susceptibility of CARD9-deficient mice to C. neoformans is due to a reduced accumulation of IFN-γ-expressing NK cells and memory phenotype T cells during the early stage of infection (6). Deficiencies in CARD9 signaling certainly lead to defects in Th1-type CD4^+^ T cell responses and IFN-γ production by innate cells. However, previous results (25–27) together with the results presented herein indicate that deficiencies in the antimicrobial activity of macrophages are likely the key effector cell defect responsible for the inability of CARD9-deficient mice to control C. neoformans infection.

The roles of specific CLRs during the induction of protective immunity against *Cryptococcus* have proven to be a challenge to determine. For example, Dectin-1, which recognizes β-glucans, and Dectin-3, which also recognizes α-mannans in *Candida* spp. are dispensable for protection against *Cryptococcus* (52, 53). However, the loss of specific CLRs may potentially be compensated for by other CLRs that recognize similar or disparate moieties on or within the pathogen. Studies to determine the roles of CLRs in mediating protection against C. neoformans are all conducted using single receptor knockout mice. The genetic challenge of generating multi-CLR KO mice is that CLR-encoding regions are located in the syntenic telomeric ends of chromosomes 6 for mice and 12 in humans and mice (54, 55). CARD9 is a critical adaptor protein that operates downstream of several immunoreceptor tyrosine-based activation motif (ITAM)-associated CLRs, including Dectin-1, Dectin-2, Dectin-3, and Mincle receptors (4). Consequently, we elected to utilize CARD9-deficient mice to study the overall impact of CLRs during the induction phase of vaccine-mediated antifungal responses.

In summary, our study shows that CARD9 is a key player for the elicitation of NO production, M1 macrophage activation, and protective anticryptococcal responses necessary for the induction of protective vaccine-mediated immune responses. Any defects in macrophage activity will have a significant deleterious impact on vaccine-mediated immune responses against C. neoformans due to the role of macrophages in mediating protective effector cell responses against C. neoformans. However, the impact may not be as hard felt in other models of infectious diseases where the role of other effector cells, such as neutrophils, are more emphatic.

## MATERIALS AND METHODS

### Mice.

Male and female CARD9 KO mice on a C57BL/6 background were a generous gift from Marcel Wüthrich (University of Wisconsin–Madison, Madison, WI), while wild-type C57BL/6 (H-2^b^) sex- and age-matched mice were purchased from the National Cancer Institute/Charles River Laboratories. All animal experiments were conducted following NIH guidelines for housing and care of laboratory animals and in accordance with protocols approved by the Institutional Animal Care and Use Committee (protocol number MU021) of the University of Texas at San Antonio. Humane endpoint by CO_2_ asphyxiation followed by cervical dislocation was conducted if death of the animals during the following hours was expected.

### Strains and media.

Cryptococcus neoformans strain H99 (serotype A, mating type α), and the P_GPD1_-*ZNF2* strain or LW10 strain (8), a generous gift from Xiaorong Lin (University of Georgia, Athens, GA) were recovered from 15% glycerol stocks stored at –80°C and maintained on yeast-peptone-dextrose (YPD) medium agar plates (Becton Dickinson, Sparks, MD). Yeast cells were grown for 16 to 18 h at 30°C with shaking in liquid YPD broth, collected by centrifugation, washed three times with sterile phosphate-buffered saline (PBS), and viable yeasts were quantified using trypan blue dye exclusion on a hemocytometer.

### Pulmonary cryptococcal infections and fungal burden.

Mice were anesthetized with 2% isoflurane utilizing a rodent anesthesia device (Eagle Eye Anesthesia, Jacksonville, FL) and were infected via the intranasal route with either 1 × 10^4^ CFU of C. neoformans strain H99 or 1 × 10^6^ CFU for strain LW10 in 50 μl of sterile PBS. The inocula used for the nasal inhalation were verified by quantitative culture on YPD agar. Mice were euthanized on predetermined days by CO_2_ inhalation followed by cervical dislocation, and lung tissues were excised. The left lobe of the lung was removed and homogenized in 1 ml of sterile PBS as previously described (27) followed by culture of 10-fold dilutions of each homogenate on YPD agar supplemented with chloramphenicol. CFU were enumerated following incubation at 30°C for 48 h. For survival studies, mice were inoculated as stated above, monitored twice daily, and sacrificed if moribund.

### Pulmonary leukocyte isolation.

The lungs of WT and CARD9 KO mice (*n* = 5/group) were excised on day 7 postinoculation as previously described (27). The lungs were then digested enzymatically at 37°C for 30 min in 10 ml digestion buffer (RPMI 1640 and 1 mg/ml collagenase type IV [Sigma-Aldrich, St. Louis, MO]) with intermittent (every 10 min) stomacher homogenizations as described in reference 56. The digested tissues were then successively filtered through sterile 70- and 40-μm nylon filters (BD Biosciences, San Diego, CA) to enrich for leukocytes, and the cells were then washed three times with sterile Hanks’ balanced salt solution (HBSS). Erythrocytes were lysed by incubation in NH_4_Cl buffer (0.859% NH_4_Cl, 0.1% KHCO_3_, 0.0372% Na_2_EDTA [pH 7.4]; Sigma-Aldrich) for 3 min on ice followed by a twofold excess of sterile PBS. T cells were first depleted by anti-CD3 antibodies and subsequent binding using antibiotin magnetic beads (Miltenyi Biotec, Auburn, CA). The leukocyte population was then enriched for pulmonary macrophages using biotinylated anti-F4/80 antibody (clone BM8; eBioscience) and subsequent binding using antibiotin magnetic beads (Miltenyi Biotec) according to the manufacturer’s recommendations and as described in reference 56. Following isolation of F4/80^+^ macrophages, the remaining cells were enriched for dendritic cells (DCs) via positive selection by labeling cells with anti-CD11c-labeled magnetic beads (Miltenyi Biotec) according to the manufacturer’s recommendations. The purity of each cell type was validated via flow cytometry using labeling check reagents (Miltenyi Biotec) with F4/80^+^ and CD45 antibodies (eBioscience) for macrophages and CD11c^+^ and CD11b^+^ antibodies for dendritic cells (the purity of macrophages and DCs was routinely >85% and >95%, respectively).

### Flow cytometry.

Standard methodology was employed for the direct immunofluorescence of pulmonary leukocytes. Briefly, 96-well U-bottom plates containing 1 × 10^6^ cells in 100 μl of PBS plus 2% fetal bovine serum (FBS) (fluorescence-activated cell sorting [FACS] buffer) were incubated with 100 μl of Fc block or CD16/32 (BD Bioscience) diluted in FACS buffer to prevent nonspecific binding of antibodies to cellular Fc receptors. Subsequently, an optimal concentration of fluorochrome-conjugated antibodies (between 0.06 and 0.5 μg per 1 × 10^6^ cells) was added, and cells were incubated for 30 min at 4°C. Following incubation, the cells were washed three times with FACS buffer and fixed in 200 μl of 2% ultrapure paraformaldehyde (Affymetrix) diluted in FACS buffer (fixation buffer). Cells incubated with either FACS buffer alone or single fluorochrome-conjugated antibodies were used to determine positive staining and spillover/compensation calculations and the flow cytometer-determined background fluorescence. The samples were analyzed using FlowJo software from a BD LRS-II flow cytometer (BD Biosciences) (56). Dead cells were excluded on the basis of forward angle and 90° light scatter. For data analyses, 100,000 events (cells) were evaluated from a predominantly leukocyte population identified by back-gating from CD45^+^-stained cells. The absolute numbers of leukocytes (CD45^+^ cells), CD4^+^/CD3^+^/CD45^+^ T cells, CD8^+^/CD3^+^/CD45^+^ T cells, CD19/CD45^+^ B cells, CD11b^+^/Ly6G^+^/CD45^+^ polymorphonuclear leukocytes (PMNs), F4/80^+^/SiglecF^+^/CD11b^−^/CD11c^+^/CD45^+^ alveolar macrophages, F4/80^+^/SiglecF^−^/CD11b^+^/CD11c^+^/CD45^+^ interstitial macrophages, CD11c^int^/CD11b^+^/CD45^+^ DCs, SiglecF^+^/CD11b^int+^/CD45^+^ eosinophils, B220^+^/CD11c^+^/PDCA-1^+^/CD45^+^ plasmacytoid DCs (pDCs), γδ^+^/CD45^+^ γδ T cells, NKp46^+^/CD45^+^ NK cells, and CD3^+^/NKp46^+^/CD45^+^ NKT cells were determined by multiplying the percentage of each gated population by the total number of CD45^+^ cells.

### Cytokine analysis.

Cytokine levels within lung tissue homogenates were analyzed using the Bio-Plex protein array system (Luminex-based technology; Bio-Rad Laboratories, Hercules, CA). Briefly, lung tissue was excised and homogenized in ice-cold sterile PBS (1 ml). An aliquot (50 μl) was taken to quantify the pulmonary fungal burden and an anti-protease buffer solution (1 ml) containing PBS, protease inhibitors, and 0.05% Triton X-100 was added to the homogenate. Samples were then clarified by centrifugation (3,500 rpm) for 10 min. Supernatants from pulmonary homogenates and *ex vivo* macrophages were assayed for the presence of interleukin IL-1α, IL-1β, IL-2, IL-4, IL-5, IL-6, IL-10, IL-12(p40), IL-12(p70), IL-13, IL-17A, keratinocyte-derived cytokine or KC (CXCL1), monocyte chemoattractant protein 1 or MCP-1 (CCL2), macrophage inflammatory protein or MIP-α (CCL3), MIP-β (CCL4), regulated upon activation normally T-expressed and presumably secreted or RANTES (CCL5), Eotaxin (CCL11), gamma interferon (IFN-γ), tumor necrosis factor alpha (TNF-α), and granulocyte-macrophage colony-stimulating factor (GM-CSF), and granulocyte colony-stimulating factor (G-CSF) according to the manufacturer’s instructions.

### Pulmonary dendritic cell and macrophage anticryptococcal killing assay.

Pulmonary F4/80^+^ macrophages and CD11c^+^ DCs were enriched from mice on day 7 postinoculation as described above, and the viability of phagocytic cells was assessed using trypan blue exclusion and a hemocytometer. DCs and macrophages were cultured at a density of 5 × 10^5^ cells per well, in triplicate, in a 96-well tissue culture plate in R10 medium as previously described (25). The initial fungal burden was analyzed by lysing phagocytes using sterile deionized water, followed by serial dilution and plating on YPD agar supplemented with chloramphenicol (Mediatech, Manassas, VA) for 48 h at 30°C. After 24 h of incubation at 37°C, macrophages and dendritic cells were lysed with water, and serial dilutions were also plated on YPD agar to enumerate CFU as described previously (56). Nitric oxide (NO) was analyzed by Griess reagent (Sigma-Aldrich) according to the manufacturer’s recommendations.

### Real-time PCR analysis.

Total RNA was isolated from enriched F4/80 cells using TRIzol reagent (Invitrogen, Carlsbad, CA) and DNase (Qiagen, Germantown, MD) treated to remove DNA contamination, according to the manufacturer’s instructions. Total RNA was subsequently recovered using Qiagen’s RNeasy kit. cDNA was synthesized from 2 ng of total RNA using oligo(dT) primer and reagents supplied by SuperScript III reverse transcriptase (RT) system (Invitrogen), according to the manufacturer’s instructions. The cDNA was used as a template for real-time PCR analysis using TaqMan gene expression assay (Applied Biosystems, Foster City, CA), according to the manufacturer’s instructions. All real-time PCRs were performed using the 7900HT Fast Real-Time PCR system (Applied Biosystems). For each real-time PCR, a master mix was prepared on ice with TaqMan gene expression assays specific for *IFN-γ*, *iNOS*, *TNF-α*, *Arg1*, *IL-4*, and *IL-13* (Applied Biosystems). TaqMan rodent glyceraldehyde-3-phosphate dehydrogenase (GAPDH) (Applied Biosystems) was used for an internal control. The thermal cycling parameters consisted of an initial denaturing cycle of 95°C for 10 min, followed by 40 cycles, with 1 cycle consisting of 95°C for 15 s and 60°C for 60 s. Real-time PCR data were derived using the comparative threshold cycle (*C_T_*) method to detect relative gene expression as described in reference 26, and statistical analysis compared the *C_T_* values of our gene of interest of CARD9 compared to WT after being normalized to murine GAPDH.

### Immunohistochemistry and histology.

Mice were sacrificed according to approved protocols, and the lungs were immediately perfused with sterile PBS by transcardial perfusion through the right ventricle. The pericardium and trachea were exposed by dissection, and an incision was made in the trachea for the insertion of a sterile flexible cannula attached to a 3-ml syringe. The lungs were then inflated with 0.5 to 0.7 ml of 10% ultrapure formaldehyde (Polysciences, Inc., Warrington, PA), excised, and immediately fixed in 10% ultrapure formaldehyde for 24 h. The lungs were then transferred to 70% ethanol and subsequently mounted into cassettes and paraffin embedded by personnel at the Histology and Immunohistochemistry Laboratory of The University of Texas Health Science Center at San Antonio. After paraffin embedding, 5-mm sections were cut and stained using hematoxylin and eosin (H&E) at McClinchey Histology Labs, Stockbridge, MI. Sections were examined by light microscopy using a Nikon microscope, and microphotographs were taken using Digital Microphotography system DFX1200 with ACT-1 software (Nikon Co, Tokyo, Japan). For measurements of cryptococcal cell and capsule size, images of multiple sections were taken by using a Keyence BZ-800 microscope with a 10× objective with a 1× digital zoom. Images were imported into a BX-X800 analyzer, and the diameter of the *Cryptococcus* was determined by the XY measuring tool. The thickness of the capsule was measured by the XY measuring tool from the cell wall of the strain to the edge of the capsule as shown in Fig. S3 in the supplemental material.

### Statistical analysis.

Survival data were analyzed by using the log rank test to detect statistically significant differences using GraphPad Prism version 8.0 for Macintosh or PC (GraphPad Software, San Diego, CA). An unpaired Student’s *t* test (two-tailed test) was used to analyze fungal burden, nitric oxide production, macrophage cytokine production, and anticryptococcal activity of macrophages by Prism (GraphPad Software). Kruskal-Wallis test with Dunn’s multiple-comparison test was used to analyze cytokine and chemokine levels and pulmonary cell populations by flow cytometry. For morphometry analysis, a two-way analysis of variance (ANOVA) with Dunnett correction was used to compare the four-group comparison to the LW10 CARD9 KO group used as a reference group.

10.1128/mBio.03005-19.4 FIG S4 Gating strategy for enriched pulmonary macrophages. Download FIG S4, PDF file, 1.4 MB. Copyright © 2020 Campuzano et al. 2020 Campuzano et al. This content is distributed under the terms of the Creative Commons Attribution 4.0 International license.

10.1128/mBio.03005-19.5 TABLE S1 Antibodies used for FACS analysis. Download Table S1, PDF file, 0.5 MB. Copyright © 2020 Campuzano et al. 2020 Campuzano et al. This content is distributed under the terms of the Creative Commons Attribution 4.0 International license.
