# Supplementary figures and images for: CARD9 Is Required for Classical Macrophage Activation and the Induction of Protective Immunity against Pulmonary Cryptococcosis
Source: mBio. 2020 Jan 7;11(1):e03005-19. doi: 10.1128/mBio.03005-19 (PMC6946806; doi:10.1128/mBio.03005-19)

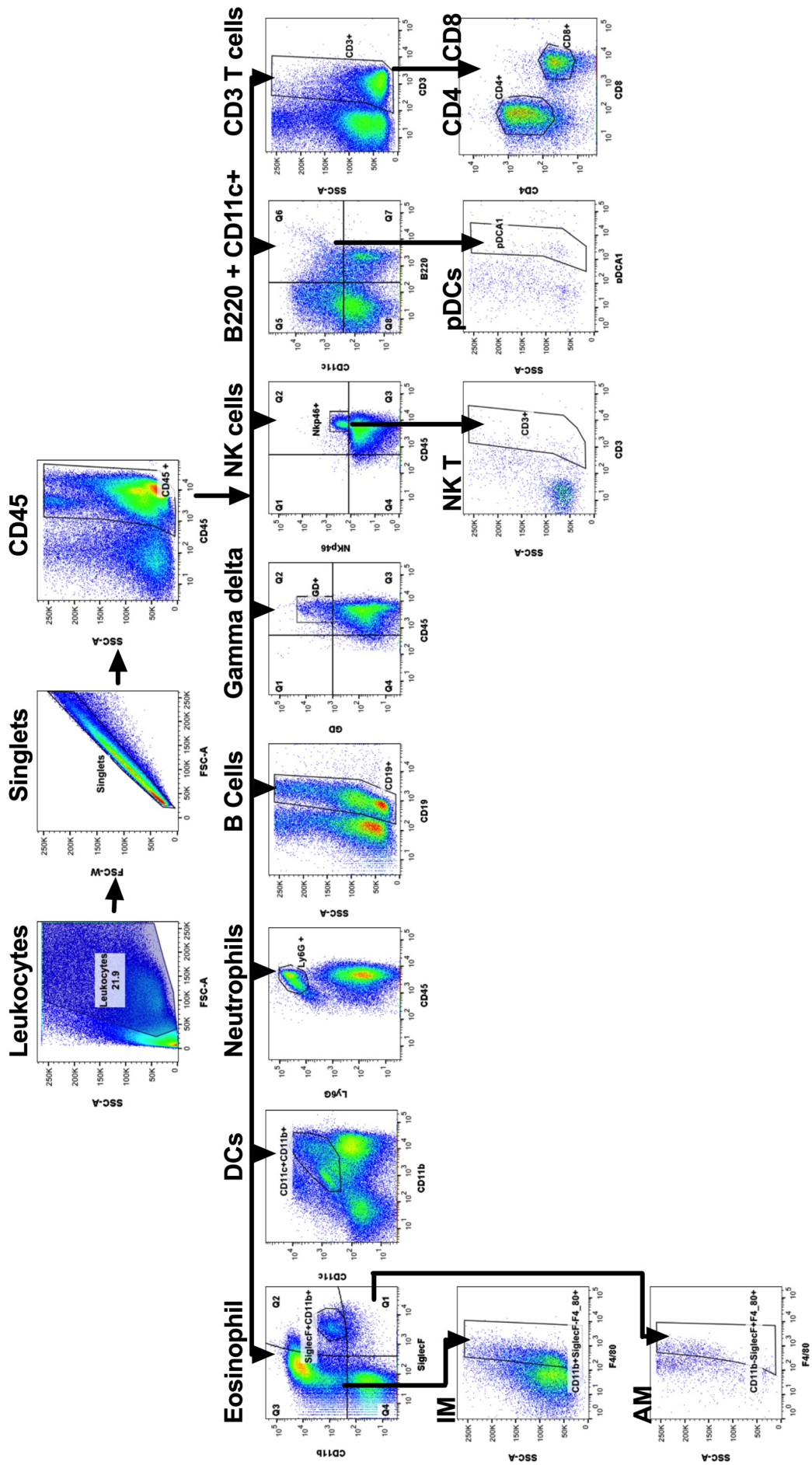

Supplement: FIG S1 [file mBio.03005-19-sf001.pdf]

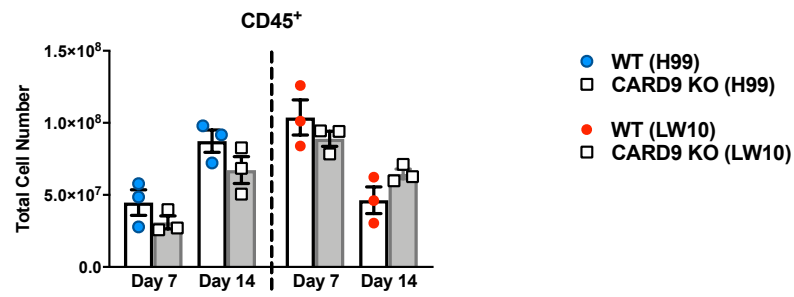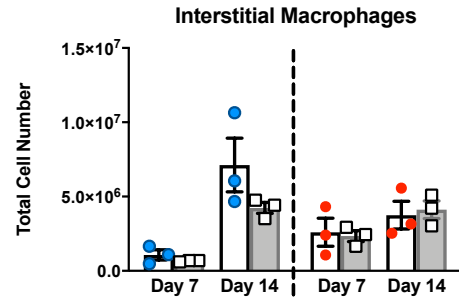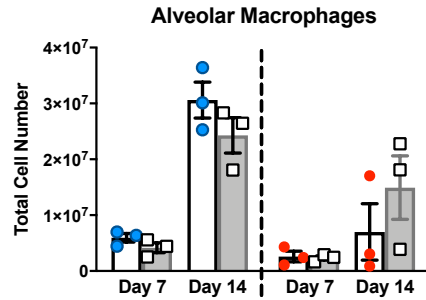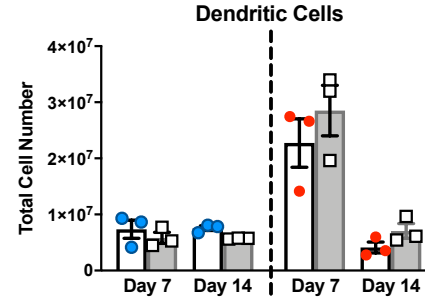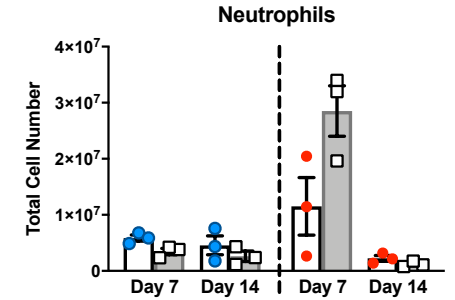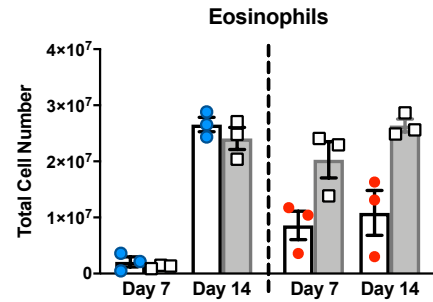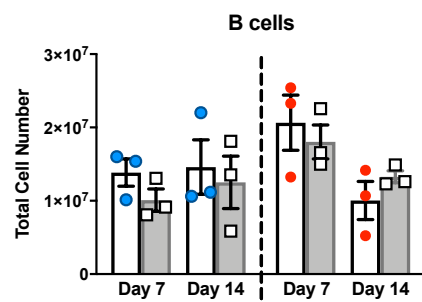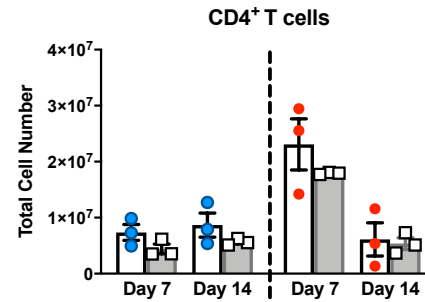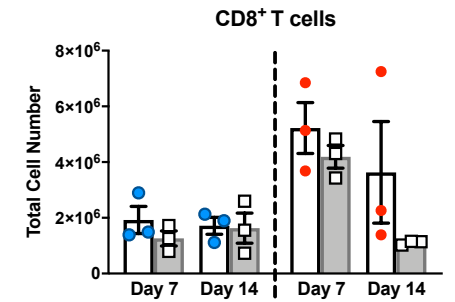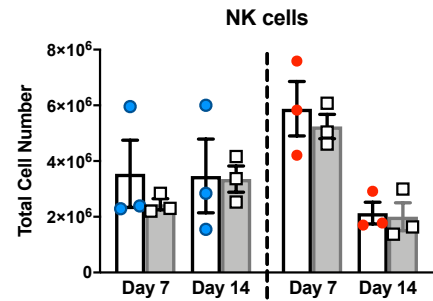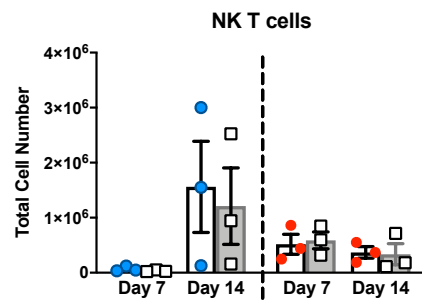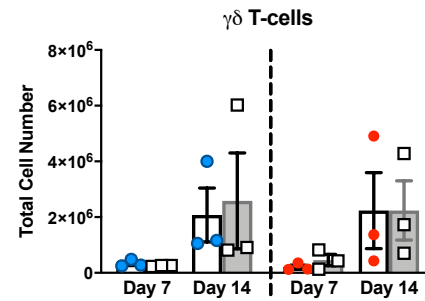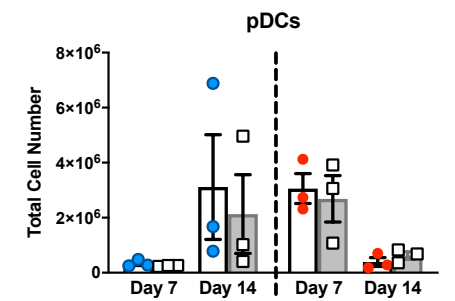

Supplement: FIG S2 [file mBio.03005-19-sf002.pdf]

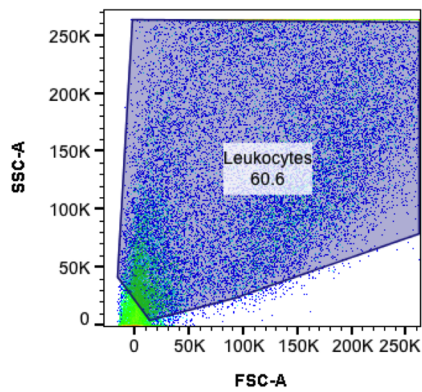

FSC-W

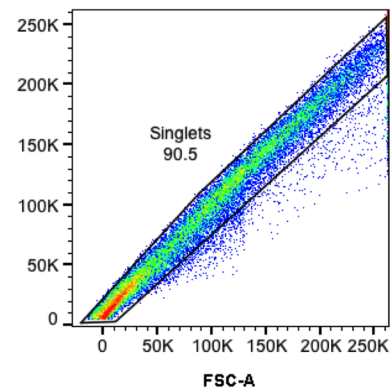

SSC-A

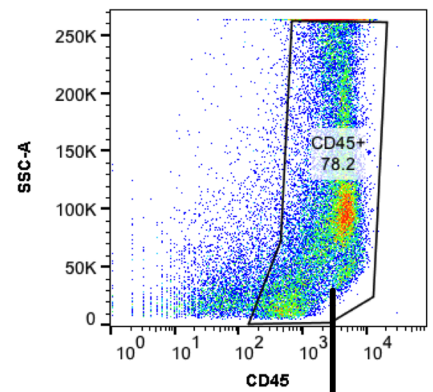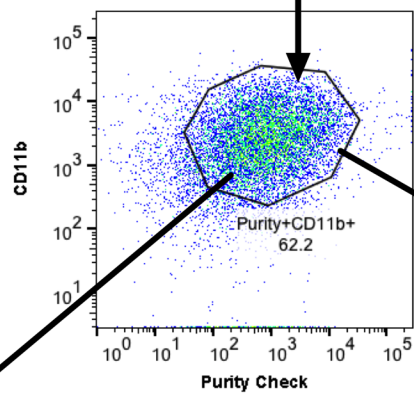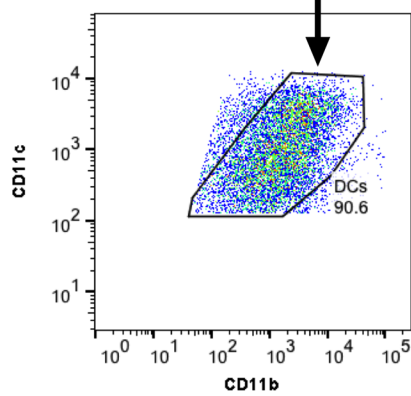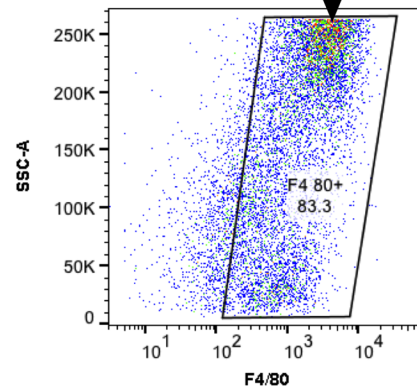

Supplement: FIG S4 [file mBio.03005-19-sf004.pdf]
