# Supplementary material for: CARD9 Is Required for Classical Macrophage Activation and the Induction of Protective Immunity against Pulmonary Cryptococcosis
Source: mBio. 2020 Jan 7;11(1):e03005-19. doi: 10.1128/mBio.03005-19 (PMC6946806; doi:10.1128/mBio.03005-19)
Supplement: FIG S3 [file mBio.03005-19-sf003.pdf]

A.

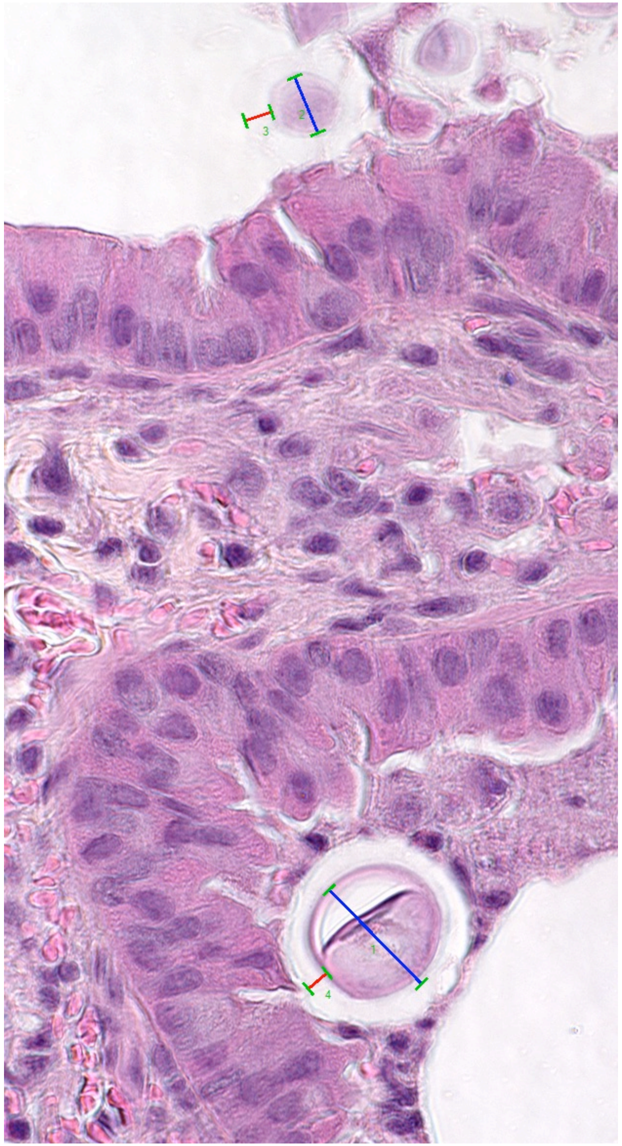

B.

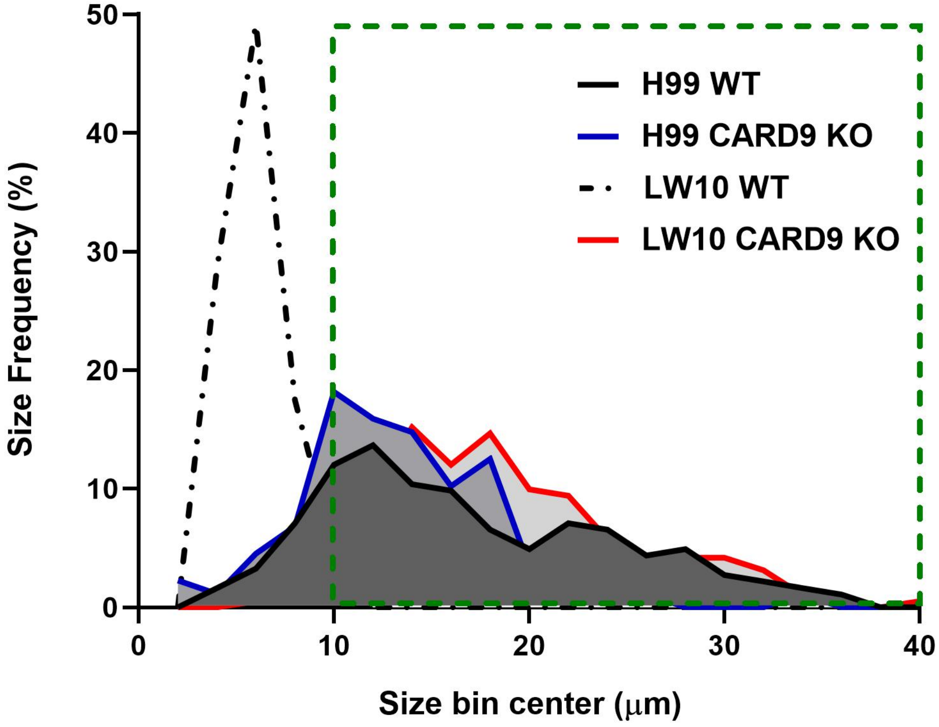

Figure S3. A) Morphometry measures for cell diameter (blue) and capsule size (red) were conducted in set of images taken from the lung sections and calculated and performed at the randomized fields (n=4 mice per group). B) Frequency plot illustrates distribution of diameters sorted using frequency distribution analysis tool in Prism 8.1 in 2 micrometers increment bins and illustrated as histograms. Green doted area represents cell size consistent with Titan Cell.
