# Supplementary material for: CARD9 Is Required for Classical Macrophage Activation and the Induction of Protective Immunity against Pulmonary Cryptococcosis
Source: mBio. 2020 Jan 7;11(1):e03005-19. doi: 10.1128/mBio.03005-19 (PMC6946806; doi:10.1128/mBio.03005-19)
Supplement: TABLE S1 [file mBio.03005-19-st001.pdf]

	Marker	Clone ID:	Fluorophore	AM	IM	DC	Neutrophil	Eosinophil	B cell	CD8	CD4	NK	NK T cell	GD	pDC
1	CD45	30-F11	FITC	+	+	+	+	+	+	+	+	+	+	+	+
2	CD11b	M1/70	PeCy7	-	+	+	+	+	+	-	-	-	-	-	-
3	CD11c	N418	PerCP-Cy5.5	+	+	+	-	-	-	-	-	-	-	-	+
4	F4/80	BM8	APC	+	+	-	-	+	-	-	-	-	-	-	-
5	SiglecF	E50-2440	BV421	+	-	-	-	+	-	-	-	-	-	-	-
6	Ly6G	1A8	PE	-	-	-	+	-	-	-	-	-	-	-	-
7	CD19	6D5	PerCP-Cy5.5	-	-	-	-	-	+	-	-	-	-	-	-
8	CD3e	145-2C11	PeCy7	-	-	±	-	-	-	+	+	-	+	+	-
9	CD4	RM4-5	FITC	-	-	±	-	-	-	-	+	-	-	+	-
10	CD8a	53-6.7	PE	-	-	±	-	-	-	+	-	-	-	+	-
11	CD335 (NKp46)	29A1.4	eFluor 660	-	-	-	-	-	-	-	-	+	+	-	-
12	Gamma Delta	GL-3	PE	-	-	-	-	-	-	-	-	-	-	-	-
13	CD45R (B220)	RA3-6B2	PeCy7	-	-	-	-	-	-	-	-	-	-	-	+
14	CD317 (pDCA-1)	eBio927	Alexa647	-	-	-	-	-	-	-	-	-	-	-	+
